# Immunogenicity of Two Doses of BNT162b2 mRNA COVID-19 Vaccine with a ChAdOx1-S Booster Dose among Navy Personnel in Mexico

**DOI:** 10.3390/v16040551

**Published:** 2024-04-01

**Authors:** Yanet Ventura-Enríquez, Evelyn Cortina-De la Rosa, Elizabeth Díaz-Padilla, Sandra Murrieta, Silvia Segundo-Martínez, Verónica Fernández-Sánchez, Cruz Vargas-De-León

**Affiliations:** 1 Banco de Sangre, Centro Médico Naval (CEMENAV), Coyoacán, Ciudad de México 04470, Mexico; yanereb@gmail.com (Y.V.-E.); elizabeth.dipad@gmail.com (E.D.-P.); murrietasandra10@yahoo.com.mx (S.M.); chivis21985@gmail.com (S.S.-M.); 2 Departamento de Hematología, Instituto Nacional de Cardiología Ignacio Chávez, Ciudad de México 14080, Mexico; evelyncortina@yahoo.com.mx; 3 Facultad de Química, Universidad Nacional Autónoma de México (UNAM), Ciudad de México 04510, Mexico; 4 Facultad de Estudios Superiores Iztacala (FES-Iztacala), Universidad Nacional Autónoma de México (UNAM), Ciudad de México 54090, Mexico; 5 División de Investigación, Hospital Juárez de México, Ciudad de México 07760, Mexico; 6 Laboratorio de Modelación Bioestadística Para la Salud, Sección de Estudios de Investigación y Posgrado, Escuela Superior de Medicina, Instituto Politécnico Nacional, Ciudad de México 11340, Mexico

**Keywords:** antibody IgG, vaccine, SARS-COV-immunity, COVID-19, spike

## Abstract

Booster doses of the SARS-CoV-2 vaccine have been recommended to improve and prolong immunity, address waning immunity over time, and contribute to the control of the COVID-19 pandemic. A heterologous booster vaccine strategy may offer advantages over a homologous approach. To compare the immunogenicity of two doses of BNT162b2 mRNA COVID-19 vaccine with a ChAdOx1-S booster dose, immunoglobulin G (IgG) anti-spike (anti-S) and anti-nucleocapsid (anti-N) antibody titers (Ab) were compared over 1 year and post-booster vaccination. Results showed that, at 3- to 9-month assessments in vaccinated subjects, an-ti-N Ab were undetectable in participants with no history of COVID-19. In contrast, anti-S Ab measurements were lower than those with COVID-19, and a decrease was observed during the 9 months of observation. After booster vaccination, no differences were found in anti-S between participants who reported a history of COVID-19 and those who did not. Anti-S levels were higher after booster vaccination measurement vs. at 9 months in participants with COVID-19 and without COVID-19, i.e., independent of an infection history. Vaccine administration elicited a response of higher anti-S IgG levels in those infected before vaccination, although levels decreased during the first nine months. IgG anti-N titers were higher in participants with a history of declared infection and who were asymptomatic. The ChAdOx1-S booster increased anti-S Ab levels in participants regardless of whether they had been infected or not to a significantly higher value than with the first two vaccines. These findings underscore the importance of booster vaccination in eliciting a robust and sustained immune response against COVID-19, regardless of the prior infection status.

## 1. Introduction

Coronavirus disease 2019 (COVID-19) has affected millions of people worldwide and was declared a pandemic by the World Health Organization (WHO) on 11 March 2020.

The urgency of a prophylactic vaccine mobilized the world to produce these vaccines. Different health authorities worldwide have developed and approved several vaccines for application [1]. In Mexico, according to the National Vaccination Policy against COVID-19, the first shipment of three thousand doses of the BNT162b2 vaccine (Pfizer-BioTech, hereafter referred to as BNT) was made available, which was, firstly, destined for health sector personnel considered as the first line of battle against COVID-19 and, secondly, for workers of essential services and people with conditions that predispose serious morbidity due to infection.

The BNT vaccine is a nucleotide-modified RNA vaccine formulated with lipid nanoparticles encoding a full-length SARS-CoV-2 S (“Spike”) protein. This vaccine underwent rigorous safety and efficacy evaluation in Germany and the US [2,3]. For any licensed vaccine, the efficacy and duration of protection are crucial. Vaccine efficacy to protect against infection above 80% is desirable. However, the duration of protection remains uncertain based on data on immunity to other coronaviruses, suggesting that immunity to SARS-CoV-2 may be short-lived or insufficient to block reinfection. They may last between 12 to 18 months [4]. It is still unclear whether a past infection will prevent severe COVID-19 upon re-exposure to SARS-CoV-2 [5,6].

Some relevant studies on the mRNA vaccine against COVID-19 have been reported [7,8,9,10,11,12,13,14]. In Korea, adverse events among healthcare workers who received all three doses of the BNT vaccine were analyzed. It was found that adverse events experienced with the first and second doses increased the incidence of adverse events at the time of the third dose. Conversely, grade 4 adverse events could still occur with the third dose, although there were no side effects with the first and second doses [7]. In the United States, a case-control study of two, three, or four doses of mRNA vaccines compared to unvaccinated adults for the Omicron BA.4 and BA.5 subvariants showed that vaccine effectiveness with three or four doses was higher compared to two doses [8].

Another study conducted a case-control study in unvaccinated and all-vaccinated military personnel in the United States. It estimated the effectiveness of mRNA-1273 (Moderna), BNT, and JNJ-78436735 (Johnson & Johnson) vaccines before and during the predominance of the Delta variant. Efficacy was significantly lower during the period of Delta variant dominance than before Delta dominance; this was especially true for the JNJ-78436735 vaccine [9]. In Bahrain, an observational study with heterologous primary booster vaccination with the BNT vaccine in those who had received two doses of the inactivated virus vaccine BBIBP-CorV (Sinopharm) demonstrated a more robust immune response against SARS-CoV-2 than the homologous BBIBP-CorV booster. It appeared to be safe and well tolerated [10].

In the UK, an observational study in adults estimated a modest benefit of booster vaccination with mRNA-1273 compared to BT162b2 in preventing positive SARS-CoV-2 tests and hospital admission with COVID-19 twenty weeks after vaccination in those who received the primary schedule of BT162b2 or adenoviral vector-based vaccine (ChAdOx1 nCoV-10, AstraZeneca, hereafter referred to as ChAd), for a period with the Delta variant followed by the Omicron variant [11]. In the United States, a cohort study of beneficiaries of the Military Health System was conducted, in which they found differential immune responses between the ChAd vaccine and the BNT-primed groups after two additional booster doses of BNT. The study showed differential humoral and cellular immune responses between the ChAd-BNT-BNT-BNT heterologous and BNT-BNT-BNT-BNT homologous vaccination cohorts [12]. 

In Thailand, a cohort study compared the immunogenicity and reactogenicity of five vaccine regimens. It found that the BNT-BNT booster induced the highest concentration of anti-receptor binding domain (anti-RBD-WT) levels. In contrast, ChAd-BTN induced the highest mean of neutralizing antibody (NAb-WT) against wild-type SARS-CoV-2. NAb-WT levels against the variants of interest, particularly the Omicron strain, were markedly attenuated for all vaccine regimens [13]. A cohort study on Military Health System beneficiaries in the United States compared humoral responses in participants vaccinated with SARS-CoV-2 infection or vaccine/infection combinations (“hybrid immunity”). Vaccine receipt elicited higher anti-spike-IgG responses than infection alone, although IgG levels declined more rapidly in vaccinated participants than in infection alone [14].

These studies suggest that, although the vaccines were associated with protection against hospitalization, one dose was not sufficient as IgG antibody titers declined in some cases between four and six months post-vaccination and a second dose and booster were necessary to increase vaccine efficacy. 

The Naval Medical Center (CEMENAV, for its acronym in Spanish) has 1475 naval personnel, including administrative and health care personnel. During the pandemic, CEMENAV was considered a COVID-19 hospital, so its personnel needed to receive the complete vaccination schedule, and they achieved 99.9% vaccination. Although the naval personnel do not represent the totality of the Mexican population, it is a controlled population that allowed us to follow up with the personnel vaccinated with the three doses of BTN and a ChAd booster in one year. We aimed to compare the immunogenicity in vaccinated personnel with or without a history of SARS-CoV-2 infection and the effect of the ChAd booster.

## 2. Materials and Methods

### 2.1. Study Design and Study Population

This is a single-center prospective cohort study conducted from March 2021 to January 2022 at the CEMENAV, Navy Secretary, Mexico City, Mexico. All personnel (administrative and health care workers (HCWs)) of CEMENAV over 18 years old who complied with the Pfizer/BioNTech BNT162b2 vaccination schedule and agreed to participate voluntarily and signed informed consent were included. The study was approved by the Research and Research Ethics Committee of the CEMENAV (approval number: 29/2021). All participants gave written informed consent before enrollment in the study. 

### 2.2. Vaccination Schedule and Vaccine Administration

This study involved navy personnel who received two doses of the Pfizer-BioTech BNT162b2 vaccine. Follow-up was completed for one year, with four blood samples, which were considered as visits: visit 1 (3 months), visit 2 (6 months), visit 3 (9 months) with the BTN vaccine, and visit 4 (1 year, 3rd booster) with the ChAd vaccine.

### 2.3. Data Collection

At the time of enrollment, participant characteristics were collected. The history of COVID-19 in participants was confirmed by RT-PCR testing, and participants without a history of COVID-19 were self-reported. Subjects who denied infection were classified as asymptomatic when they had anti-nucleocapsid levels greater than or equal to 1.4 Index.

### 2.4. Blood Collection

Venous whole blood was obtained in vacuum tubes without anticoagulant (Beckton Dickinson, Franklin Lakes, NJ, USA) to obtain serum. The samples were centrifuged at 1500× *g* for 15 min at room temperature.

### 2.5. Serological Testing

Serum was used to measure anti-nucleocapsid IgG (Abbott Diagnostics^®^, Chicago, IL, USA) and anti-S1/S2 antibodies to SARS-CoV-2 (DiaSorin^®^, Saluggia, Italy). These are described below.

#### 2.5.1. IgG Nucleocapsid

The serum obtained was tested in an Abbott Architect i4000SR (Abbott Diagnostics^®^), where SARS-CoV-2 IgG assay was performed per the manufacturer’s instructions. The assay is a chemiluminescent immunoassay that detects IgG raised against the nucleocapsid protein of SARS-CoV-2. A signal/cut-off (S/CO) ratio of ≥1.4 was interpreted as reactive and an S/CO ratio of <1.4 was interpreted as non-reactive. Calibration was performed, and positive quality control (QC) S/CO 1.65–8.40 and negative quality control S/CO ≤ 0.78 were fulfilled before analyses of patient samples.

#### 2.5.2. IgG S1/S2 Spike Protein

The LIAISON SARS-CoV-2 S1/S2 IgG antibody (quantitative assay) was performed on a Liaison-XL (DiaSorin^®^). The cut-off was >15.0 AU, and it includes a negative (<3.8 AU) and a positive (>31.9 AU) control. The assay was performed according to the manufacturer’s instructions.

### 2.6. SARS-CoV-2 Detection for Real-Time Reverse Transcriptase-Polymerase Chain Reaction (RT-PCR)

RT-PCR was performed from nasopharyngeal samples, and the extraction of total genetic material from navy personnel was performed with the QIAamp Viral RNA kit (Qiagen, Hilden, Germany), using the QIAcube-classics kit (QIAcube-classics, Qiagen, Hilden, Germany). The amplification of specific genes (Rd, Rp, E, N) for SARS-CoV-2 was performed using a GeneFinder COVID19 PLUS realAmp kit, and qRT-PCR was performed using the Applied Biosystems 7500 FAST kit (Applied Biosystems, Waltham, MA, USA). All samples were inactivated in a class A-II biosafety cabinet following the Biosafety and Good Laboratory Practice protocols issued by the WHO and the Institute of Epidemiological Diagnosis and Reference (InDRE, for its acronym in Spanish) in Mexico. 

### 2.7. Statistical Analysis

A descriptive analysis (mean and standard deviation for numerical variables and absolute and relative frequency for categorical variables) was performed to summarize the study sample’s demographic characteristics, comorbidities, and addictions.

To explore whether participants with and without a history of COVID-19 were homogeneous, Student’s *t*-test and chi-square test with and without continuity correction were used.

We performed log transformation for the nucleocapsid and spike levels to obtain a normal data distribution.

Nucleocapsid and spike comparisons between the two groups in repeated measurements were performed using repeated measures ANOVA. Analyses included fixed effects for time (3, 6, 9 months and ChAd-booster), COVID-19 history groups (present and absent), and the time × COVID-19 interaction. If the interaction was statistically significant, a post hoc analysis was performed using Sidak’s correction method for means differences to determine whether changes in nucleocapsid and spike levels between longitudinal assessments were statistically significant among participants reporting the presence or absence of COVID-19. When necessary, a Greenhouse–Geisser correction was applied to correct for nonsphericity.

Paired samples Student’s *t*-test was used to compare nucleocapsid and spike levels between 9 months and the ChAd-booster.

All analyses employed IBM Statistics SPSS 21, while line graphs of two group data sets were performed in GraphPad Prism. Statistical significance was reached at a value of *p* < 0.05.

## 3. Results

### 3.1. Description of the Study Population

**Figure 1:**
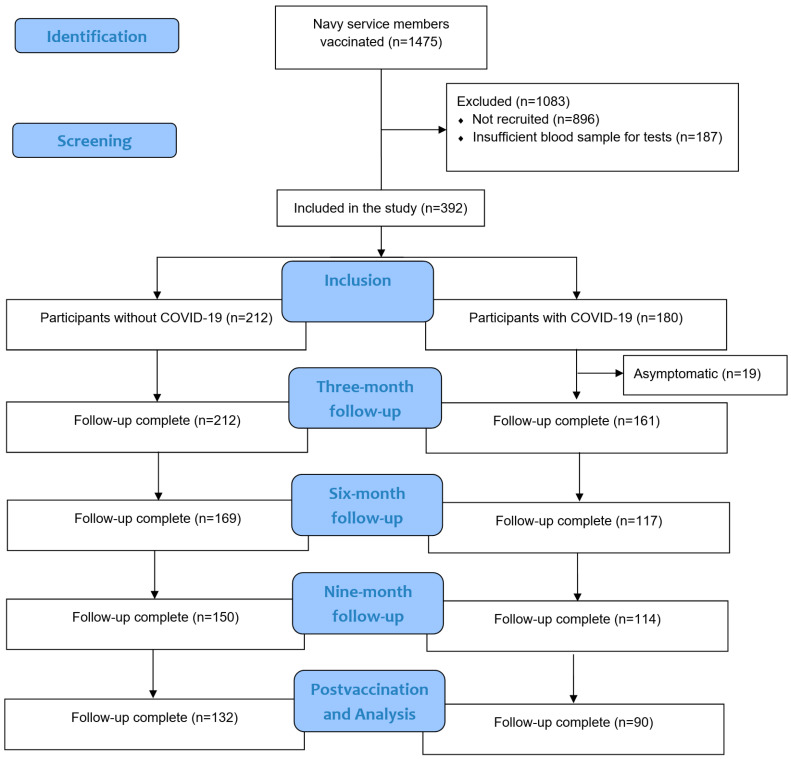
STROBE Flow diagram.

The results are presented for 392 volunteer military personnel at the time of sampling on four occasions: at 3, 6, and 9 months after receiving the two doses of mBT162b2 mRNA and one month after receiving the third booster with ChAdOx1-S Astra Zeneca. There was a total of 212 participants without COVID-19 history and 231 with COVID-19 history. Of the participants with COVID-19 history, 161 were symptomatic cases, and 19 were asymptomatic cases (Figure 1). We enrolled 373 subjects, 191 men (51.2%) and 182 women (48.8%). Median, minimum, and maximum age values in years were as follows: 36.0 (20.0–82.0).

Table 1 shows that the age between subjects with and without a COVID-19 history was not statistically different (*p*-value = 0.053), 35.7 versus 37.4 years old, respectively. The distribution of sex is homogeneous between the groups (*p*-value = 0.610). Also, the diabetes, hypertension, allergy, alcoholism, and smoking were homogeneous. Alternatively, the group with a history of COVID-19 presented 23 participants with obesity compared to the group that did not have a history of COVID-19, where 14 were found (*p*-value = 0.022).

### 3.2. Antibodies of SARS-CoV-2 Anti-N and Anti-S 

Table 2 presents the descriptive data performed without missing values of the logarithms of anti-N and anti-S to longitudinal measurements and COVID-19 history. 

Table 3 shows the repeated measures ANOVA analysis for the logarithms of anti-N and anti-S, performed without missing data. For the analysis of logarithms of anti-N, there were 114 participants with COVID-19 and 150 without COVID-19 up to nine months of follow-up, and for logarithms of anti-S, there were 58 participants with COVID-19 and 97 without COVID-19 post-vaccination. The analyses revealed significant effects for both the main variables (time and COVID-19) and the interaction (time × COVID-19), indicating that there would be differences in anti-N and anti-S levels between participants who did or did not have COVID-19.

**Figure 2:**
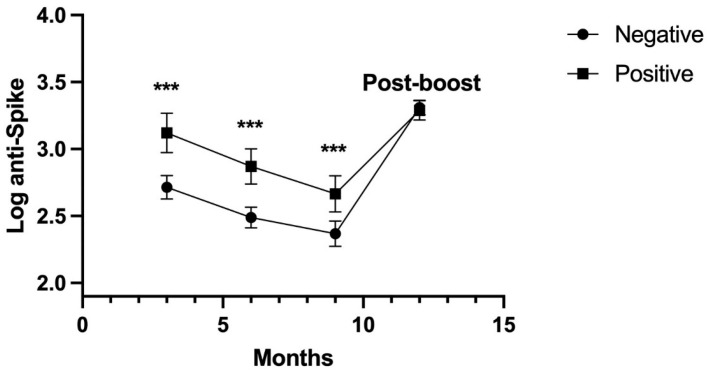
Anti-S levels during one year of observation. Anti-S levels before booster with Astra Zeneca vaccine are shown to decrease. *** *p* < 0.001. Values expressed as the means ± 2 SEM were evaluated by repeated measures ANOVA.

The repeated measures analysis of log anti-N and anti-S levels showed a statistical difference between participants who reported a history of COVID-19 and those who did not. For log anti-N, the results are expressed as a mean (standard deviation), at three months: −0.350 (0.749) versus −1.195 (0.594), *p*-value < 0.001; at six months: −0.526 (0.714) versus −1.214 (0.532), *p*-value < 0.001; and at nine months: −0.668 (0.702) versus −1.129 (0.552), *p*-value < 0.001. At the 3- to 9-month assessments, the anti-N value was higher in participants with COVID-19. Alternatively, in subjects with a positive history of COVID-19, the post-vaccination baseline anti-S value was higher than in the negative history of COVID-19; the values in both groups decreased over the 9 months. The value of anti-S antibodies in positive and negative subjects increased post-booster (Figure 2).

Anti-S levels were higher at post-booster versus the 9-month value in participants with COVID-19 (−0.624 [95%CI: −0.758, −0.490], *p*-value < 0.001) and without COVID-19 (−0.941 [95%CI: −1.069, −0.813], *p*-value < 0.001).

Table 4 compares anti-N and anti-S antibodies between participants with and without a COVID-19 history, considering the number of participants in each evaluation through cohort follow-up. The estimates of the mean and standard deviation shown in Table 4 are consistent with those without missing data in Table 3. On the other hand, the analysis of comparisons of means between two groups was carried out independently for each evaluation, and we found similar results to the post hoc analysis of the interaction in Table 2 and Table 3.

### 3.3. Asymptomatic

Nineteen participants declared not having presented COVID-19; however, the anti-N titer was higher than the cut-off point (1.4); in other words, they were asymptomatic participants. Of these, 8 (41.1%) were women and 11 (57.9%) were men. The anti-N levels at 3, 6, and 9 months were 0.428 (0.179), −0.285 (0.918), and −0.421 (0.773), respectively. The levels of anti-S at 3, 6, and 9 months and the ChAd-booster were 2.957 (0.345), 2.699 (0.446), 2.531 (0.388), and 3.227 (0.213), respectively. The last three anti-N and anti-S observations were with 15 participants. No comparisons were made with participants with or without COVID-19 as the sample was small and was shrinking over time.

## 4. Discussion

This study presents the immunogenicity of two doses of BNT162b2 mRNA COVID-19 vaccine with a ChAd booster dose in a cohort study of the vaccination program among navy personnel initiated in Mexico from 2021 to 2022. A total of 99.9% of the navy personnel completed their vaccination schedule, and 100% with at least one dose. Following booster vaccination, there were no differences in anti-S levels between participants with or without a history of COVID-19. However, anti-S levels significantly increased the ChAd-booster compared to levels at 9 months, irrespective of prior infection status. Notably, individuals previously infected exhibited higher anti-S IgG levels after vaccination, although these levels declined within the first nine months. Participants with a history of asymptomatic infection also displayed elevated IgG anti-N titers. The ChAdOx1-S booster substantially elevated anti-S antibody levels in all participants, surpassing levels achieved with the initial two vaccine doses. 

Our findings extend those of previous studies on hybrid immunity and show that the increase in anti-S IgG levels is greater in participants with a history of COVID-19. This latter finding is consistent with data showing that infections before vaccination significantly increase immunogenicity [14,15,16]. In a study with health workers, they found that previous infection with COVID-19 elicits a stronger antibody response, particularly targeting the spike receptor binding domain, six months post-vaccination, compared to individuals without prior clinical COVID-19 experience [16]. Still, anti-S IgG levels after booster vaccination are equal, regardless of the COVID-19 history. It is suggested that using a booster dose mimics COVID-19 disease in people without a COVID-19 history of increased spike antibody levels.

As expected, antibodies against the nucleocapsid do not increase significantly due to the vaccine administration [17,18]. However, nucleocapsid antibodies showed a different path compared between Navy personnel with and without a history of COVID-19, due to a previous infection.

Although it was not the purpose of our study, we found the presence of fifteen asymptomatic cases of COVID-19 among the study participants, which represents 8.5% of the cases with infections, as demonstrated by the high titers of anti-N that exceed the established cut-off point. We report summary measures of anti-N and anti-S antibody levels, but this is insufficient for statistical analysis, warranting further investigation into the dynamics of long-term immunity.

A recent systematic review found robust immunogenicity and tolerable reactogenicity of heterologous administration of a BNT162b2 boost in ChAdOx1-primed participants [19]. Several studies have documented increased immunogenicity from ChAd booster vaccination [19,20,21]. In a clinical trial, they studied the immunogenicity with a prime vaccination and booster with the BTN and ChAd vaccines (with the following combinations: prime-BTN/post-BTN, prime-BTN/post-ChAd, prime-ChAd/post-ChAd, and prime- ChAd/post-BTN); they found that all four regimens studied induced SARS-CoV-2 anti-spike IgG concentrations at least as high as those induced after a licensed prime-ChAd/post-ChAd regimen. However, the BNT-containing schemes were more immunogenic than the homologous prime-ChAd/post-ChAd scheme. Cellular immune responses in regimens containing the BNT vaccine were also at least as high as those in the prime-ChAd/post-ChAd group, with prime-BTN/post-ChAd showing the greatest expansion of vaccine-antigen responsive T cells in the peripheral circulation at 28 days after the boost [20]. COVID-19 vaccine effectiveness declines over time and can be temporarily restored with a booster dose. Heterologous boosting with BNT162b2 is more immunogenic than homologous boosting in adults primed with various non-mRNA COVID-19 vaccines but is also more reactogenic [21]. A clinical trial of third-dose booster vaccines administered 10 to 12 weeks after an initial course of ChAd/ChAd or BNT/BNT COVID-19 immunization showed the potential of all vaccines tested (ChAd, BNT, mRNA1273, NVX-CoV2373 [Novavax, hereafter referred to as NVX], Ad26, CVnCov [CureVac, hereafter referred to as CVn], and VLA2001 [Valneva, hereafter referred to as VLA]) to boost immunity after an initial course of ChAd/ChAd and of six vaccines (ChAd, BNT, mRNA1273, NVX, Ad26, and CVn) after an initial course of BNT/BNT [22]. The results of our study align with recent systematic reviews and clinical trials, highlighting the potential of heterologous boosting strategies in restoring vaccine effectiveness over time. Further research is warranted to elucidate booster vaccinations’ long-term efficacy and safety in diverse populations.

One significant drawback is our study’s observational methodology, which naturally limits our capacity to infer causal relationships between variables. Furthermore, the scope of our study is restricted to a particular subset of Navy personnel, which may constrain the applicability of our conclusions to other demographic contexts. Additionally, we only evaluated humoral immune responses; we neglected to evaluate cellular immunological responses, which may shed light on the effectiveness of vaccines and the operation of the immune system. Another limitation of the study was that the booster did not evaluate anti-nucleocapsid antibodies. Furthermore, our findings’ statistical power and accuracy may be constrained by our study population’s relatively small sample size. Additionally, we had missing participants during the follow-up period, which may have introduced bias and affected the robustness of our findings.

## 5. Conclusions

The findings of this study emphasize the significant role of heterologous vaccination in eliciting a strong antibody response against the spike protein of SARS-CoV-2, irrespective of the prior infection status.

## Figures and Tables

**Table 1:** Demographic characteristics and comorbidities of those vaccinated.

	COVID-19		
Clinical Characteristics	Present (n = 161)	Absent (n = 212)	*p*-Value
Age (Years) *	35.7 (8.18)	37.4 (8.50)	0.053
Sex (Male) **	80 (49.7%)	111 (52.4%)	0.610
Obesity **	23 (14.3%)	14 (6.60%)	**0.022**
Diabetes **	0 (0.0%)	2 (0.9%)	0.603 ***
Hypertension **	4 (2.5%)	5 (2.4%)	0.937 ***
Allergy **	1 (0.6%)	3 (1.4%)	0.818 ***
Alcoholism **			0.446
Never	28 (17.6%)	29 (14.0%)	
Occasionally	129 (81.1%)	177 (85.5%)	
Three times per week	2 (1.3%)	1 (0.5%)	
Smoking **			0.702
Never	107 (67.3%)	139 (67.5%)	
Occasionally	48 (30.2%)	62 (30.2%)	
1–5 cigarettes a day	3 (1.9%)	5 (2.4%)	
6–15 cigarettes a day	1 (0.6%)	0 (0.0%)	

* Mean (SD); SD, Standard Deviation; ** n (%); *** Yates’s Correction. Significant *p*-values are in bold.

**Table 2:** Antibodies of SARS-CoV-2 anti-N and anti-S in vaccinated participants were performed without missing data.

	COVID-19	3 Months M (SD)	*p*-Value *	6 Months M (SD)	*p*-Value *	9 Months * M (SD)	*p*-Value *
**log (anti-Nucleocapsid)**	**Present**	−0.350 (0.749)	**<0.001**	−0.526 (0.714)	**<0.001**	−0.668 (0.702)	**<0.001**
	**Absent**	−1.195 (0.594)		−1.214 (0.532)		−1.129 (0.552)	
**log (anti-Spike)**	**Present**	3.073 (0.508)	**<0.001**	2.848 (0.454)	**<0.001**	2.630 (0.466)	**<0.001**
	**Absent**	2.696 (0.392)		2.475 (0.344)		2.349 (0.422)	

M (SD): mean value (standard deviation). * Sidak post hoc test to interaction. Significant *p*-values are in bold.

**Table 3:** Results of repeated measures ANOVA for anti-N and anti-S.

	Time			COVID-19			Interaction		
	F	df	*p*-Value	F	df	*p*-Value	F	df	*p*-Value
log(anti-nucleocapsid) *	6.98	1.862	0.001	75.374	1	<0.001	14.87	1.86	**<0.001**
log(anti-Spike) *	164.96	2.527	<0.001	33.01	1	<0.001	11.88	2.527	**<0.001**

* with a Greenhouse–Geisser correction. F: a value of the F statistic; df: degrees of freedom. Significant *p*-values are in bold.

**Table 4:** Antibodies of SARS-CoV-2 anti-N and anti-S in vaccinated participants through cohort follow-up.

	COVID-19	3 Months M (SD)	*p*-Value *	6 Months * M (SD)	*p*-Value *	9 Months * M (SD)	*p*-Value *
**log (anti-Nucleocapsid)**	**Present**	n = 161	**<0.001**	n = 117	**<0.001**	n = 114	**<0.001**
		−0.338 (0.734)		−0.509 (0.714)		−0.615 (0.698)	
	**Absent**	n = 212		n = 169		n = 150	
		−1.196 (0.568)		−1.188 (0.558)		−1.103 (0.580)	
**log (anti-Spike)**	**Present**	n = 161	**<0.001**	n = 117	**<0.001**	n = 114	**<0.001**
		3.000 (0.525)		2.804 (0.434)		2.643 (0.453)	
	**Absent**	n = 212		n = 169		n = 150	
		2.644 (0.379)		2.453 (0.333)		2.343 (0.411)	

M (SD): mean value (standard deviation). * Student’s *t*-test. Significant *p*-values are in bold.

## Data Availability

The data sets used to support the findings of this study are available from the corresponding author upon reasonable request.
